# Deferasirox demonstrates a dose-dependent reduction in liver iron concentration and consistent efficacy across subgroups of non-transfusion-dependent thalassemia patients

**DOI:** 10.1002/ajh.23445

**Published:** 2013-04-04

**Authors:** Ali T Taher, John B Porter, Vip Viprakasit, Antonis Kattamis, Suporn Chuncharunee, Pranee Sutcharitchan, Noppadol Siritanaratkul, Renzo Galanello, Zeynep Karakas, Tomasz Lawniczek, Dany Habr, Jacqueline Ros, Yiyun Zhang, M Domenica Cappellini

**Affiliations:** 1 Hematology and Oncology, Department of Internal Medicine, American University of BeirutBeirut, Lebanon; 2 UCL Cancer Institute, Department of Haematology, University College LondonLondon, United Kingdom; 3 Department of Pediatrics and Internal Medicine, Siriraj Hospital, Mahidol UniversityBangkok, Thailand; 4 First Department of Pediatrics, University of AthensAthens, Greece; 5 Ramathibodi Hospital, Mahidol UniversityBangkok, Thailand; 6 Chulalongkorn University and King Chulalongkorn Memorial HospitalBangkok, Thailand; 7 Ospedale Regional MicrocitemieCagliari, Italy; 8 Istanbul University, Istanbul Medical FacultyIstanbul, Turkey; 9 Novartis Pharma AGBasel, Switzerland; 10 Novartis PharmaceuticalsEast Hanover, New Jersey; 11 Department of Internal Medicine, Università di Milano, Ca Granda Foundation IRCCSMilan, Italy

## Abstract

The 1-year THALASSA study enrolled 166 patients with various non-transfusion-dependent thalassemia (NTDT) syndromes, degrees of iron burden and patient characteristics, and demonstrated the overall efficacy and safety of deferasirox in reducing liver iron concentration (LIC) in these patients. Here, reduction in LIC with deferasirox 5 and 10 mg/kg/day starting dose groups is shown to be consistent across the following patient subgroups—baseline LIC/serum ferritin, age, gender, race, splenectomy (yes/no), and underlying NTDT syndrome (β-thalassemia intermedia, HbE/β-thalassemia or α-thalassemia). These analyses also evaluated deferasirox dosing strategies for patients with NTDT. Greater reductions in LIC were achieved in patients dose-escalated at Week 24 from deferasirox 10 mg/kg/day starting dose to 20 mg/kg/day. Patients who received an average actual dose of deferasirox >12.5–≤17.5 mg/kg/day achieved a greater LIC decrease compared with the ≥7.5–≤12.5 mg/kg/day and >0–<7.5 mg/kg/day subgroups, demonstrating a dose–response efficacy. LIC reduction across patient subgroups was generally consistent with the primary efficacy analysis with a similar safety profile. Am. J. Hematol. 88:503–506, 2013. © 2013 Wiley Periodicals, Inc.

## Introduction

Non-transfusion-dependent thalassemias (NTDTs) are a group of inherited hemoglobin disorders—including β-thalassemia intermedia (β-TI), mild or moderate forms of HbE/β-thalassemia, and α-thalassemia (including HbH disease) which affect patients of different genders, ages, and genetic backgrounds. Disease severity can vary widely among individuals and management strategies often differ depending on clinical complications 1. Over time, patients with NTDT are at risk of clinically relevant iron overload, attributed mainly to increased intestinal iron absorption due to ineffective erythropoiesis 2. Although patients do not require regular red blood cell transfusions to survive, occasional blood transfusions may be needed for growth failure or during pregnancy and infections 3–7, which may exacerbate iron overload. Factors affecting the rate or the amount of iron overload have been identified in patients with NTDT. Management modality such as splenectomy has been associated with marked increases in iron loading 8–10. Other factors influencing iron loading are age 11, disease genotype, and different ethnic/genetic backgrounds, determined by variants in iron-regulating genes 12–15.

Recently, elevated liver iron concentration (LIC) and serum ferritin levels in patients with β-TI have been linked with an increased risk of vascular, endocrine, and bone disease 11,16. Patients with NTDT have minimal or no dependence on transfusions; however, there is still a requirement for effective iron chelation therapy in patients with NTDT to prevent serious complications relating to iron overload. Patients enrolled in the recent THALASSA (assessment of Exjade® in non-transfusion-dependent THALASSemiA patients) study had different underlying NTDT syndromes and presented with variable ages, genders, ethnic backgrounds, baseline levels of iron overload, and previous history of splenectomy. The variation in these parameters in patients with NTDT and the effect they have on response to iron chelation therapy are of interest; hence, these analyses evaluate the trial primary efficacy endpoint of reducing LIC after one year treatment with deferasirox in the different subgroups.

## Patients and Methods

The 1-year, randomized, double-blind, placebo-controlled THALASSA study (ClinicalTrials.gov number NCT00873041) evaluated iron chelation therapy in patients with NTDT and showed that deferasirox (Exjade) compared to placebo significantly reduces iron overload in these patients with an overall good safety and tolerability profile 17. Patients with a variety of NTDT syndromes (β-TI, HbE/β-thalassemia, or α-thalassemia), degrees of iron burden, and clinical characteristics were included in this study; therefore, additional analyses of patient subgroups were performed to examine the consistency of deferasirox efficacy and safety amongst this diverse population of patients. Deferasirox dose could be doubled after Week 24 in patients with insufficient response (LIC >7 mg Fe/g dw and reduction <15% compared with baseline) 17. Absolute change from baseline in LIC (as measured by FerriScan® 18) was analyzed by age, gender, ethnic group, splenectomy (yes/no), dosing pattern (escalated/non-escalated), actual dose received, baseline LIC, baseline serum ferritin, and underlying NTDT syndrome. In addition, safety parameters were also analyzed according to these patient subgroups. The primary efficacy analysis was performed via the analysis of covariance (ANCOVA), including the treatment as factor and baseline LIC as covariate. Least squares mean (LSM) estimates were obtained from the ANCOVA model for absolute change in LIC from baseline to Week 52 (last observation carried forward). The LSM differences between treatment arms and their 95% confidence intervals were calculated with Dunnett's adjustment for multiplicity. Similar to the primary analysis, the ANCOVA model was fitted and the LSM estimates with corresponding 95% confidence intervals are presented for each subgroup separately.

## Results

In total, 166 patients with β-TI (*n* = 95), HbE/β-thalassemia (*n* = 49), or α-thalassemia (*n* = 22) were randomized in a 2:1:2:1 ratio to deferasirox 5 mg/kg/day (*n* = 55)/matching placebo (*n* = 28) starting dose or 10 mg/kg/day (n=55)/matching placebo (*n* = 28) starting dose, as previously described 17.

### Age, gender, race, splenectomy, and underlying NTDT syndrome

The primary endpoint was analyzed by subgroup (age, gender, race, baseline LIC categories, baseline serum ferritin categories, splenectomy status, and underlying NTDT syndrome) using ANCOVA to explore the consistency of treatment effects found overall. In all subgroups, there was a trend for greater reduction in LIC in all patients treated with deferasirox compared with the placebo group, particularly in patients receiving deferasirox 10 mg/kg/day starting dose (Supporting Information Table 1). This mirrors observations seen in the whole population, where at Week 52, LIC decreased from baseline by LSM ± SE of −1.95 ± 0.50 mg Fe/g dw and −3.80 ± 0.48 mg Fe/g dw in the deferasirox 5 and 10 mg/kg/day starting dose groups, and increased by 0.38 ± 0.49 mg Fe/g dw in the overall placebo group 17.

### Analysis by subgroups

**Figure 1:**
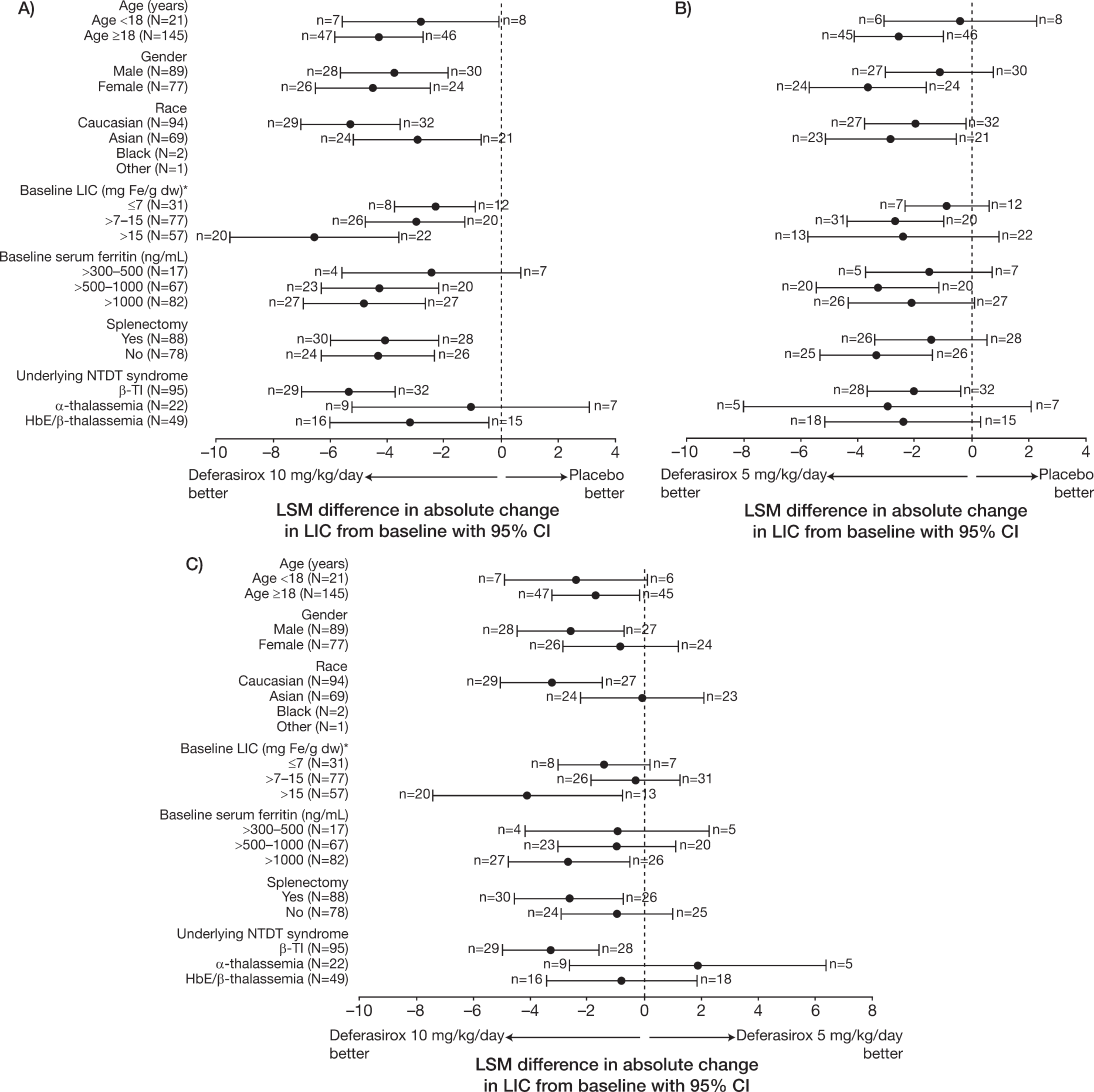
Forest plots of LSM with 95% confidence interval (CI) for differences in absolute change in LIC from baseline to Week 52 between (A) deferasirox 10 mg/kg/day starting dose and all placebo groups combined; (B) deferasirox 5 mg/kg/day starting dose and placebo groups; and (C) deferasirox 5 mg/kg/day and deferasirox 10 mg/kg/day starting dose groups. *n = 1 missing.

In general, patients receiving deferasirox 5 mg/kg/day and 10 mg/kg/day starting doses had better LIC responses in the majority of subgroups compared with placebo as shown by the point estimates of the differences favoring the deferasirox 10 mg/kg/day and 5 mg/kg/day starting dose arms (Fig. 1A,B). Within the deferasirox arms, patients in the deferasirox 10 mg/kg/day starting dose group had better LIC responses than those in the deferasirox 5 mg/kg/day starting dose group (Fig. 1C).

### Analysis by baseline LIC and baseline serum ferritin

**Figure 2:**
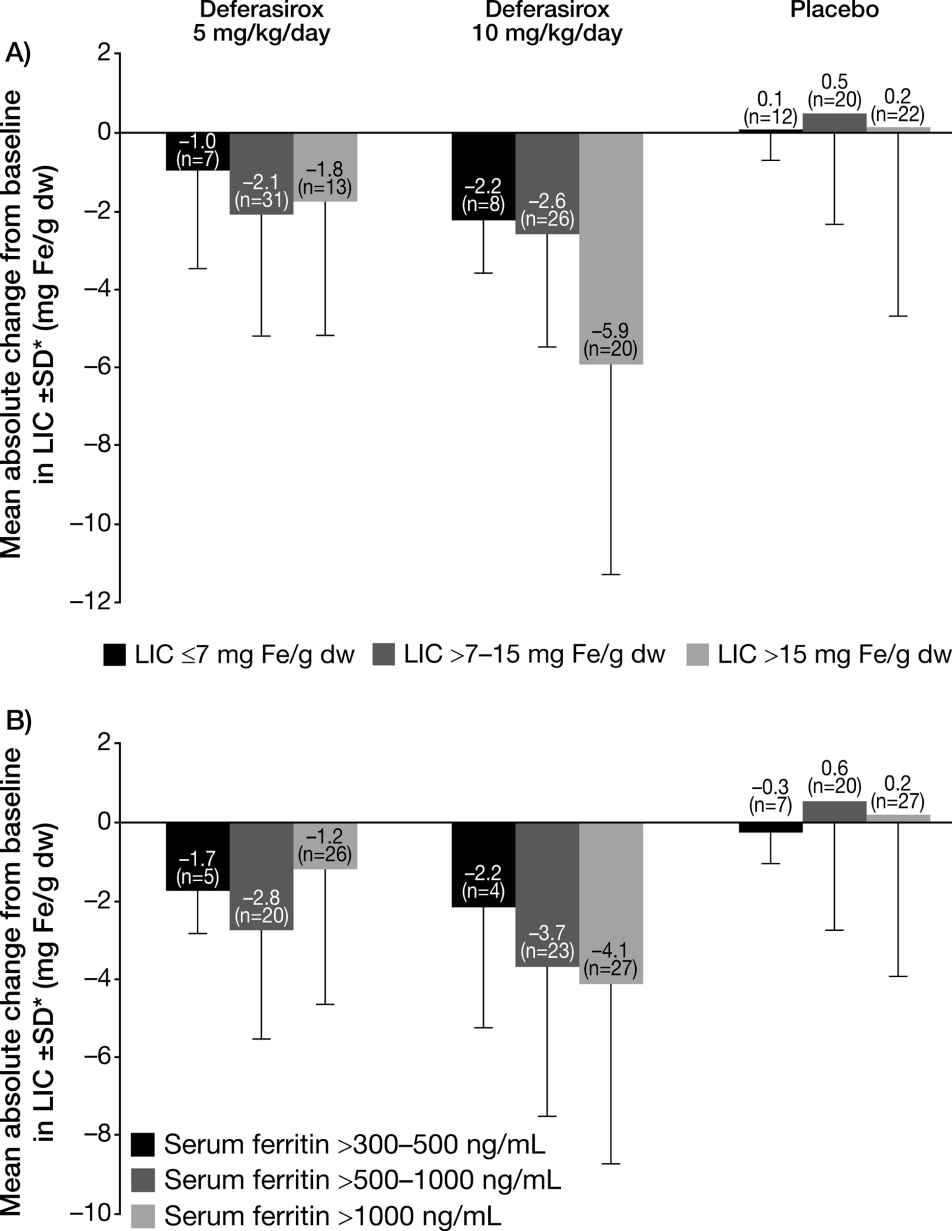
Mean absolute change from baseline in LIC to Week 52 ± SD by (A) baseline LIC category; and (B) baseline serum ferritin category. *The last available post-baseline LIC was carried forward if no LIC value was available at Week 52.

Patients in the deferasirox 10 mg/kg/day starting dose group with baseline LIC >15 mg Fe/g dw had a greater reduction in LIC at Week 52 (−5.9 ± 5.3 mg Fe/g dw [*n* = 20]) than those with baseline LIC >7–15 mg Fe/g dw (−2.6 ± 2.9 mg Fe/g dw [*n* = 26]) or baseline LIC ≤7 mg Fe/g dw (−2.2 ± 1.3 mg Fe/g dw [*n* = 8]). For patients in the deferasirox 5 mg/kg/day starting dose group, LIC reduction was comparable for baseline LIC >15 mg Fe/g dw (−1.8 ± 3.4 mg Fe/g dw [*n* = 13]), LIC >7–15 mg Fe/g dw (−2.1 ± 3.1 mg Fe/g dw [*n* = 31]) and LIC ≤7 mg Fe/g dw (−1.0 ± 2.5 mg Fe/g dw [*n* = 7]; Fig. 2A).

Reduction in LIC was also observed in all baseline serum ferritin subgroups (>1,000, >500–1,000, and >300–500 ng/mL) for patients treated in both deferasirox starting dose groups. Reductions in LIC at Week 52 for patients in the deferasirox 10 mg/kg/day starting dose group were greater in the >1,000 ng/mL (baseline: 18.1 ± 7.9 mg Fe/g dw; Week 52: 13.8 ± 7.2 mg Fe/g dw) baseline serum ferritin subgroup compared with the >500–1,000 ng/mL (baseline: 10.8 ± 6.5 mg Fe/g dw; Week 52: 7.1 ± 7.0 mg Fe/g dw) and >300–500 ng/mL (baseline: 11.4 ± 4.7 mg Fe/g dw; Week 52: 9.3 ± 6.6 mg Fe/g dw) subgroups. Absolute change in LIC was −4.1 ± 4.6 mg Fe/g dw [*n* = 28], −3.7 ± 3.8 mg Fe/g dw [*n* = 23] and −2.2 ± 3.1 mg Fe/g dw [*n* = 4], for >1,000, >500–1,000, and >300–500 ng/mL baseline serum ferritin subgroups, respectively; Fig. 2B).

### Dosing pattern (escalated/non-escalated and actual average daily dose received)

Overall, the 81 patients who received escalated doses had a greater mean baseline LIC (15.6, 17.6 and 19.0 mg Fe/g dw in the deferasirox 5 mg/kg/day, 10 mg/kg/day and placebo starting dose groups, respectively) compared with those who remained on their starting dose (10.9, 12.0, and 12.3 mg Fe/g dw in the deferasirox 5 mg/kg/day, 10 mg/kg/day and placebo starting dose groups, respectively; Supporting Information Fig. 1).

Mean ± SD LIC reduction was greater for patients whose dose was escalated from deferasirox 10 to 20 mg/kg/day compared with patients maintained on deferasirox 10 mg/kg/day starting dose throughout the study (−4.0 ± 4.8 mg Fe/g dw and −3.6 ± 3.5 mg Fe/g dw, respectively) (actual mean ± SD dose received 14.1 ± 2.0 mg/kg/day and 9.3 ± 1.4 mg/kg/day, respectively). LIC reduction for patients receiving deferasirox 5 mg/kg/day starting dose with or without escalation to 10 mg/kg/day was similar (−1.8 ± 3.1 mg Fe/g dw and −1.9 ± 3.1 mg Fe/g dw, respectively) (mean ± SD actual deferasirox dose received 6.8 ± 1.0 and 4.6 ± 0.7 mg/kg/day, respectively; Supporting Information Fig. 1).

In addition, patients were stratified according to the actual dose received. The reduction in LIC was the greatest in patients who received deferasirox at an average actual dose >12.5–≤17.5 mg/kg/day (−4.2 ± 5.1 mg Fe/g dw [*n* = 20]), followed by the ≥7.5–≤12.5 mg/kg/day subgroup (−3.6 ± 3.4 mg Fe/g dw [*n* = 37]) and the >0–<7.5 mg/kg/day subgroup (−1.7 ± 3.2 mg Fe/g dw [*n* = 48]) demonstrating a clear dose–response effect.

### Safety overview

The most common investigator-assessed drug-related adverse events (AEs) in the THALASSA trial were nausea, skin rash, diarrhea, headache, upper abdominal pain, and abdominal pain (for full details see Ref.^17^). Although the number of AEs was too small to allow firm conclusions, no clinically relevant differences were observed in the deferasirox AE profile across the analyzed subgroups. Of the eight patients who experienced AEs resulting in study discontinuation (*n* = 3 deferasirox 5 mg/kg/day starting dose group, *n* = 3 deferasirox 10 mg/kg/day starting dose group, *n* = 2 placebo 10 mg/kg/day starting dose group), all were aged >18 years, five were female and seven were Caucasian. Laboratory evaluations (shifts in alanine aminotransferase, serum creatinine, creatinine clearance, urinary protein/creatinine ratios, or change in hematology variables from baseline) also did not show relevant differences across subgroups.

## Discussion

NTDTs include a range of thalassemia syndromes with variable disease severities which may complicate diagnosis and treatment. Iron overload and associated complications are a common observation in patients with NTDT, particularly as patients age even though they require limited or no transfusions 11. Effective, timely management of iron overload is key in patients with NTDT to prevent iron-related complications. The THALASSA study is the largest study assessing iron chelation in patients with NTDT, thus allowing for the analyses of the individual subgroups within the NTDT population to be made. The ANCOVA analyses indicate that the efficacy of deferasirox is consistent across the patient subgroups, including the NTDT subtypes such as β-TI and HbE/β-thalassemia, which will have varying levels of disease severity. In some subgroups, such as patients less than 18 years and those with α-thalassemia, the patient numbers were low and confidence intervals (CIs) were wide; hence data should be interpreted with caution. LIC was reduced in a dose-dependent manner in accordance with the primary efficacy outcome, with a greater reduction seen in the 10 mg/kg/day starting dose group compared with the 5 mg/kg/day starting dose group.

In addition to assessing subgroups, these analyses investigated the initial impact of dose escalation of deferasirox to 20 mg/kg/day in patients with NTDT. This is important given that many NTDT patients have a significantly high iron burden, hence the need for dose adjustments to achieve therapeutic goals similar to transfusion-dependent thalassemia patients. Here, following initiation of treatment with either placebo, deferasirox 5 or 10 mg/kg/day starting dose, 81 out of 166 patients (49%) required a dose escalation. These patients had a greater mean baseline LIC compared with those who remained on their starting dose. Patients who had their dose doubled to 20 mg/kg/day achieved greater reductions in LIC compared with patients who remained on the 10 mg/kg/day starting dose. The reduction in LIC by average actual dose also showed that patients who received deferasirox >12.5–≤17.5 mg/kg/day achieved the greatest LIC decrease, followed by the ≥7.5–≤12.5 mg/kg/day subgroup and the >0–<7.5 mg/kg/day subgroup demonstrating a clear dose–response efficacy. Change in LIC was more pronounced in patients with higher baseline LIC which may reflect either higher dose of deferasirox used or a potentially increased molecular efficiency of the chelator in severely iron overloaded patients. As widely evidenced in transfusion-dependent thalassemias, optimal control of patients' iron burden is achievable through appropriate monitoring and dose adjustment of chelation therapy 19. As such, it is important to begin to understand the nuances of appropriate management of the non-transfusion-dependent population so that these patients can also achieve their therapeutic goals. This study used a fixed deferasirox starting dose of 5 or 10 mg/kg/day regardless of baseline LIC, with subsequent dose adjustments. Given that many patients with NTDT have a high degree of iron burden, it would be interesting to explore higher deferasirox starting doses in patients with higher LIC, for example those with LIC ≥15 mg Fe/g dw, in future trials. It will also be of interest to determine potential mechanisms, such as the role of ascorbate and other factors that could explain why some patients may not respond as well to iron chelation therapy 20. Clinical studies in both transfusion-dependent and non-transfusion-dependent thalassemia patients are required to further investigate such hypotheses.

Alongside deferasirox efficacy, it is of importance to determine the safety profile across the various subgroups. The safety profile for these NTDT patients treated with deferasirox is consistent across the subgroups with no clinically relevant differences.

The THALASSA 1-year extension study will provide further insight into the efficacy and safety profile of deferasirox as well as optimal dosing strategies for patients with NTDT, with further dose adjustments permitted.
