# Supplementary material for: Deferasirox demonstrates a dose-dependent reduction in liver iron concentration and consistent efficacy across subgroups of non-transfusion-dependent thalassemia patients
Source: Am J Hematol. 2013 Apr 4;88(6):503–6. doi: 10.1002/ajh.23445 (PMC3698696; doi:10.1002/ajh.23445)
Supplement: Supplementary file 1 [file ajh0088-0503-SD1.doc]

**Supplementary data**

**Participating centers and investigators:** MD Cappellini, Ca Granda Foundation IRCCS, Milan, Italy; ME Lai, Ospedale Regionale Microcitemie, Cagliari, Italy; P Cianciulli, Fondaz. Policlinico Tor Vergata, Roma, Italy; R Galanello, Ospedale Regionale Microcitemie, Cagliari, Italy; S Perrotta, A Osp-Univ Policl Seconda, Napoli, Italy; G Forni, SSD Centro Microcitemie e Anemie Congenite, Genova, Italy; E Vichinsky, Children’s Hospital Oakland, Oakland, United States; P Giardina, Weill Cornell Medical College, New York, United States; A Thompson, Children’s Memorial Hospital, Chicago, United States; C Kattamis, ‘Agia Sofia’ Children’s Hospital of Athens, Athens, Greece; M Athanassiou-Metaxa, ‘Hippokration’ General Hospital of Thessaloniki, Thessaloniki, Greece; M Karakantza, University Hospital of Patra-RIO, Patras-RIO, Greece; JB Porter, University College Hospital, London, United Kingdom; F Shah, Whittington Hospital, London, United Kingdom; SL Thein, Kings College Hospital, London, United Kingdom; Y Aydinok, Ege University Medical Faculty Hospital, Izmir, Turkey; Z Karakas, Istanbul University Istanbul Medical Faculty, Istanbul, Turkey; F Gumruk, Hacettepe University Medical Faculty, Ankara, Turkey; Y Kilinc, Cukurova University Medical Faculty, Adana, Turkey; CL Lee, University Malaya Medical Centre, Kuala Lumpur, Malaysia; HM Ibrahim, Hospital Kuala Lumpur, Kuala Lumpur, Malaysia; ARA Jamal, Pusat Perubatan UKM, Kuala Lumpur, Malaysia; J Sathar, Jalan Mewah Utara, Ampang Selong, Malaysia; A Chuansumrit, Ramathibodi Hospital, Bangkok, Thailand; S Chuncharunee, Ramathibodi Hospital, Bangkok, Thailand; N Siritanaratkul, Siriraj Hospital, Bangkok, Thailand; V Viprakasit, Siriraj Hospital, Bangkok, Thailand; P Sutcharitchan, Chulalongkorn Hospital, Bangkok, Thailand; AT Taher, American University of Beirut, Beirut, Lebanon; K-H Lin, National Taiwan University, Taipei, Taiwan.
