# Supplementary material for: Deferasirox demonstrates a dose-dependent reduction in liver iron concentration and consistent efficacy across subgroups of non-transfusion-dependent thalassemia patients
Source: Am J Hematol. 2013 Apr 4;88(6):503–6. doi: 10.1002/ajh.23445 (PMC3698696; doi:10.1002/ajh.23445)
Supplement: Supplementary file 3 [file ajh0088-0503-SD3.doc]

***Supplementary*** Table 1. Absolute change from baseline LIC to Week 52 at last-available post-baseline visit by age, gender, race, splenectomy status and underlying NTDT syndrome

|  | **Mean LIC ±SD, mg Fe/g dw** | | | | | | | | |
| --- | --- | --- | --- | --- | --- | --- | --- | --- | --- |
|  | **Deferasirox 5 mg/kg/day starting dose** | | | **Deferasirox  10 mg/kg/day starting dose** | | | **Placebo 5 and 10 mg/kg/day starting dose** | | |
| **Subgroups** | **Baseline** | **Week 52*** | **Absolute change from baseline*** | **Baseline** | **Week 52*** | **Absolute change from baseline*** | **Baseline** | **Week 52*** | **Absolute change from baseline*** |
| **Age** | | | | | | |  |  |  |
| <18 years | 10.3 ±3.3 (n=6) | 8.7 ±4.4  (n=6) | –1.6 ±2.0 (n=6) | 11.4 ±4.5 (n=7) | 7.1 ±2.9  (n=7) | –4.3 ±3.2 (n=7) | 6.8 ±1.8  (n=8) | 6.7 ±2.2  (n=8) | –0.2 ±1.3 (n=8) |
| ≥18 years | 13.5 ±7.6 (n=49) | 11.9 ±8.2  (n=45) | –1.9 ±3.2 (n=45) | 15.0 ±8.2 (n=48) | 11.1 ±8.0  (n=47) | –3.7 ±4.3 (n=47) | 17.5 ±11.0 (n=47) | 18.1 ±10.6  (n=46) | 0.3 ±3.8 (n=46) |
| **Gender** | | | | | | |  |  |  |
| Male | 13.8 ±8.8 (n=29) | 13.4 ±9.7  (n=27) | –1.1 ±3.0 (n=27) | 13.8 ±7.9 (n=29) | 9.8 ±7.5  (n=28) | –3.6 ±4.0 (n=28) | 18.6 ±12.1 (n=30) | 18.5 ±11.7  (n=30) | –0.2 ±3.6 (n=30) |
| Female | 12.3 ±5.1 (n=26) | 9.5 ±4.6  (n=24) | –2.7 ±3.0 (n=24) | 15.4 ±8.1 (n=26) | 11.4 ±7.9  (n=26) | –4.0 ±4.4 (n=26) | 12.7 ±8.2 (n=25) | 13.8 ±8.6  (n=24) | 0.8 ±3.3 (n=24) |
| **Race** | | | | | | |  |  |  |
| Caucasian | 12.2 ±6.1 (n=31) | 11.3 ±6.8  (n=27) | –1.4 ±3.5 (n=27) | 12.7 ±6.6 (n=30) | 7.6 ±4.9  (n=29) | –4.7 ±3.6 (n=29) | 14.0 ±9.0 (n=32) | 14.4 ±9.5  (n=32) | 0.4 ±3.2 (n=32) |
| Asian | 14.5 ±8.7 (n=23) | 12.2 ±9.2  (n=23) | –2.3 ±2.5 (n=23) | 17.1 ±9.0 (n=24) | 14.4 ±8.9  (n=24) | –2.6 ±4.6 (n=24) | 19.3 ±12.6 (n=22) | 19.9 ±11.6  (n=21) | 0.02 ±4.0 (n=21) |
| Black | 8.3 (n=1) | 4.9  (n=1) | –3.4 (n=1) | 10.4 (n=1) | 5.1  (n=1) | –5.3 (n=1) | – | – | – |
| Other | – | – | – | – | – | – | 6.0 (n=1) | 5.6  (n=1) | –0.4 (n=1) |
| **Splenectomy** | | | | | | |  |  |  |
| Yes | 13.6 ±7.7 (n=29) | 13.0 ±8.7  (n=26) | –1.0 ±2.9 (n=26) | 14.6 ±7.2 (n=31) | 10.6 ±6.7  (n=30) | –3.7 ±4.1 (n=30) | 15.5 ±10.1 (n=28) | 15.9 ±10.3  (n=28) | 0.4 ±3.7 (n=28) |
| No | 12.6 ±6.9 (n=26) | 10.1 ±6.9  (n=25) | –2.8 ±3.0 (n=25) | 14.5 ±8.9 (n=24) | 10.6 ±8.9  (n=24) | –3.9 ±4.3 (n=24) | 16.4 ±11.7 (n=27) | 17.0 ±11.1  (n=26) | 0.2 ±3.3 (n=26) |
| **Underlying NTDT syndrome** | | | | | | |  |  |  |
| β-TI | 12.0 ±6.1 (n=32) | 11.2 ±6.7  (n=28) | –1.2 ±3.2 (n=28) | 13.0 ±6.5 (n=30) | 8.0 ±4.6  (n=29) | –4.5 ±3.6 (n=29) | 13.8 ±8.9 (n=32) | 14.5 ±9.~~4~~  (n=32) | 0.7 ±2.8 (n=32) |
| α-thalassemia^†^ | 11.2 ±2.3 (n=5) | 6.5 ±3.0  (n=5) | –4.7 ±2.5 (n=5) | 15.0 ±9.5 (n=9) | 12.5 ±11.8  (n=9) | –2.5 ±4.1 (n=9) | 18.8 ±12.9 (n=8) | 19.6 ±13.9  (n=7) | –0.9 ±4.0 (n=7) |
| HbE/β-thalassemia | 15.7 ±9.5 (n=18) | 13.6 ±10.0  (n=18) | –2.1 ±2.6 (n=18) | 17.3 ±9.2 (n=16) | 14.1 ±8.1  (n=16) | –3.2 ±5.0 (n=16) | 19.0 ±13.0 (n=15) | 18.9 ±11.3  (n=15) | –0.2 ±4.5 (n=15) |

*The last-available post-baseline LIC measurement was carried forward if no LIC value was available at Week 52; **^†^**HbH disease (n=8), HbH Constant Spring (n=6), genotype not determined (n=6), CSEA Barts (n=1), Hb Agrino (n=1).

***
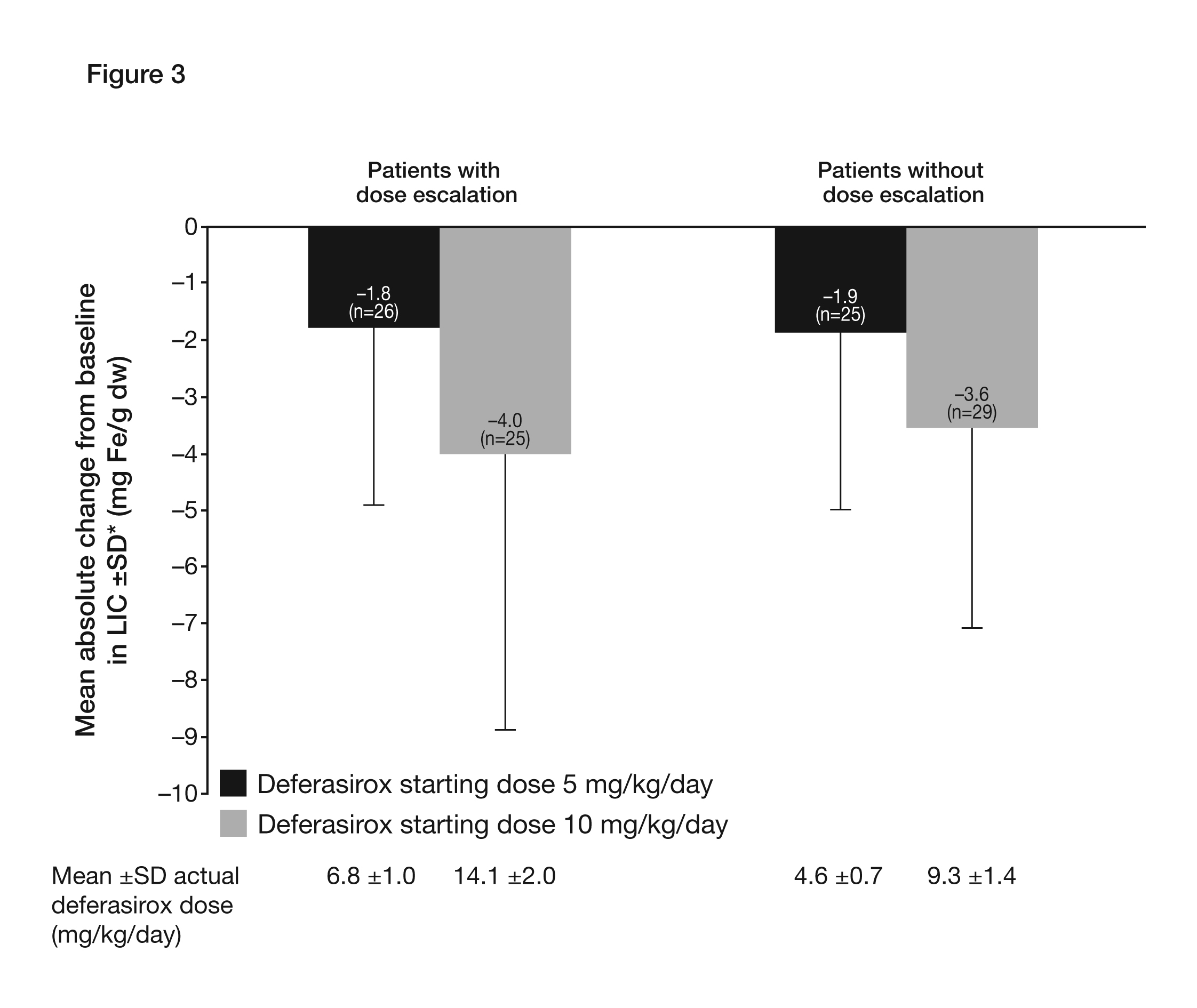
***

***Supplementary Figure 1***

*The last available post-baseline LIC was carried forward if no LIC value was available at Week 52
